# Exploring the role of pain as an early predictor of category 2 pressure ulcers: a prospective cohort study

**DOI:** 10.1136/bmjopen-2016-013623

**Published:** 2017-01-20

**Authors:** Isabelle L Smith, Sarah Brown, Elizabeth McGinnis, Michelle Briggs, Susanne Coleman, Carol Dealey, Delia Muir, E Andrea Nelson, Rebecca Stevenson, Nikki Stubbs, Lyn Wilson, Julia M Brown, Jane Nixon

**Affiliations:** 1 Clinical Trials Research Unit, Leeds Institute of Clinical Trials Research, University of Leeds, Leeds, UK; 2 Leeds Teaching Hospitals NHS Trust, Leeds, UK; 3 Division of Nursing, Midwifery & Social Work, & Central Manchester University Hospitals NHS Foundation Trust (CMFT), Faculty of Biology, Medicine and Health, University of Manchester, Manchester, UK; 4 School of Health & Population Sciences, University of Birmingham, Birmingham, UK; 5 School of Healthcare, University of Leeds, Leeds, UK; 6 Leeds Institute of Clinical Trials Research, University of Leeds, Leeds, UK; 7 Department of Tissue Viability, Leeds Community Healthcare NHS Trust, Leeds, UK; 8 Mid Yorkshire Hospital NHS Trust, Wakefield, UK

**Keywords:** Pressure Ulcers, Cohort, Pain, Observational, Risk factors, Multi-level

## Abstract

**Objective:**

To explore pressure area related pain as a predictor of category ≥2 pressure ulcer (PU) development.

**Design:**

Multicentre prospective cohort study.

**Setting:**

UK hospital and community settings.

**Participants inclusion:**

Consenting acutely ill patients aged ≥18 years, defined as high risk (Braden bedfast/chairfast AND completely immobile/very limited mobility; pressure area related pain or; category 1 PU).

**Exclusion:**

Patients too unwell, unable to report pain, 2 or more category ≥2 PUs.

**Follow-up:**

Twice weekly for 30 days.

**Primary and secondary outcome measures:**

Development and time to development of one or more category ≥2 PUs.

**Results:**

Of 3819 screened, 1266 were eligible, 634 patients were recruited, 32 lost to follow-up, providing a 602 analysis population. 152 (25.2%) developed one or more category ≥2 PUs. 464 (77.1%) patients reported pressure area related pain on a healthy, altered or category 1 skin site of whom 130 (28.0%) developed a category ≥2 PU compared with 22 (15.9%) of those without pain. Full stepwise variable selection was used throughout the analyses. (1) Multivariable logistic regression model to assess 9 a priori factors: presence of category 1 PU (OR=3.25, 95% CI (2.17 to 4.86), p<0.0001), alterations to intact skin (OR=1.98, 95% CI (1.30 to 3.00), p=0.0014), pressure area related pain (OR=1.56, 95% CI (0.93 to 2.63), p=0.0931). (2) Multivariable logistic regression model to account for overdispersion: presence of category 1 PU (OR=3.20, 95% CI (2.11 to 4.85), p<0.0001), alterations to intact skin (OR=1.90, 95% CI (1.24 to 2.91), p=0.0032), pressure area related pain (OR=1.85, 95% CI (1.07 to 3.20), p=0.0271), pre-existing category 2 PU (OR=2.09, 95% CI (1.35 to 3.23), p=0.0009), presence of chronic wound (OR=1.66, 95% CI (1.06 to 2.62), p=0.0277), Braden activity (p=0.0476). (3) Accelerated failure time model: presence of category 1 PU (AF=2.32, 95% CI (1.73 to 3.12), p<0.0001), pressure area related pain (AF=2.28, 95% CI (1.59 to 3.27), p<0.0001). (4) 2-level random-intercept logistic regression model: skin status which comprised 2 levels (versus healthy skin); alterations to intact skin (OR=4.65, 95% CI (3.01 to 7.18), p<0.0001), presence of category 1 PU (OR=17.30, 95% CI (11.09 to 27.00), p<0.0001) and pressure area related pain (OR=2.25, 95% CI (1.53 to 3.29), p<0.0001).

**Conclusions:**

This is the first study to assess pain as a predictor of category ≥2 PU development. In all 4 models, pain emerged as a risk factor associated with an increased probability of category ≥2 PU development.

**Strengths and limitations of this study**

- The study was designed to incorporate key quality criteria for the conduct and reporting of risk factor/prognostic factor studies to promote generalisability and minimise bias.
- The primary outcome, the development of new category ≥2 pressure ulcers (PUs), provides the most reliable outcome measure of PU development.
- Patients were recruited from hospital and community settings and are representative of UK ‘standard care’.
- There was no blinded outcome assessment due to funding and/or capacity within the clinical research teams.
- It is acknowledged that the patient population is not representative of the general NHS population through exclusion of patients who had cognition problems, were very sick or were terminally ill.

## Introduction

### Background

A systematic review of health-related quality of life in patients with pressure ulcers (PUs) identified that patients reported experiencing pain at ‘pressure areas’ prior to clinical PU manifestation but that their reports of pain were ignored. Patients blamed healthcare professionals when a PU developed subsequently, due to the lack of action.^1^ A PU risk factor systematic review did not identify any studies which included pain as a candidate risk factor in multivariable analysis,^2^ and previous cross-sectional studies have focused on pain associated with existing category ≥2 PUs.^3–5^

As part of the National Institute for Health Research (NIHR) funded PU Programme of Research (PURPOSE),^6^ we first sought to determine the extent of pain experienced by patients with intact skin on pressure areas.^7^ ^8^ We conducted two large multicentre prevalence surveys in the hospital^8^ and community^7^ setting. It was identified that 12.6% (233/1769) of hospital patients with no observable PUs reported pressure area related pain. In addition, substudies involving patients who consented to a detailed pain assessment, 157 hospital and 37 community patients reported pain on 68.0% (66/97) and 19/20 (95%) skin sites assessed as grade 1 (ie, intact skin with non-blanching erythema).^7^ ^8^

The results indicated the extent of pressure area related pain in hospital inpatients with skin assessed as ‘normal’ or ‘non-blanching but intact’.

In the second stage of the programme, we explored the role of pressure area related pain of intact skin as a predictor of PU development in acute hospital and community populations.

### Aims and objectives

The primary aim was to explore the role of pressure area related pain as an early predictor of category ≥2 PU development in patients at high risk of PU development. Objectives were to:

1. Assess whether the presence/absence of pressure area related pain is a predictor of category ≥2 PU development.
2. Identify variables which are independently predictive of category ≥2 PU development.

## Methods

### Study design

We conducted a prospective cohort study involving acutely ill hospital and community patients at high risk of PU development. Follow-up was twice weekly for a maximum of 30 days from registration or until they were no longer at high risk of PU development or transferred to a non-participating centre or death.

### Setting

Patients were recruited from 26 hospital and community centres across 18 NHS Trusts in England between 26 October 2009 and 17 November 2011. All centres had PU prevention and management policies and guidelines based on national and international guidelines^9^ including risk assessment and mattress provision and patients received care as determined by the attending clinical teams.

### Patients

All patients were aged ≥18 years, provided written informed consent, were able to report the presence/absence of pressure area related pain, were at high risk of PU development and indicated as acutely ill.

High risk of PU development was defined as one or more of the following: (1) bedfast/chairfast and completely immobile/have very limited mobility according to the Braden Scale,^10^ (2) localised skin pain on any pressure area skin site and (3) category 1 PU on any pressure area skin site.^2^ ^4^ ^11^

Indicators of acute illness were defined pragmatically as one of the following: acute hospital admission to vascular, orthopaedic, medical or care of the elderly specialties;^2^ ^11^ new community nursing referral following hospital discharge to home/intermediate/community care/hospice/specialist palliative care; existing community nursing patient with deterioration in overall condition or onset of acute illness; new referral to community nursing due to acute illness, deterioration in existing condition or care package breakdown.

Patients were not eligible to participate if they were admitted to obstetrics, paediatrics, day case surgery or psychiatric wards. Patients were also not eligible if they were deemed by the attending healthcare professional to be too unwell to be approached and/or complete the study assessment schedule, or they had ≥2 existing category ≥2 PUs on the following pressure area skin sites: sacrum, buttocks, heels and hips.

Eligible patients were recruited by the research team and after written informed consent/witnessed verbal consent they were registered to the study using a central 24-hour telephone registration system.

### Outcomes

**Figure 1:**
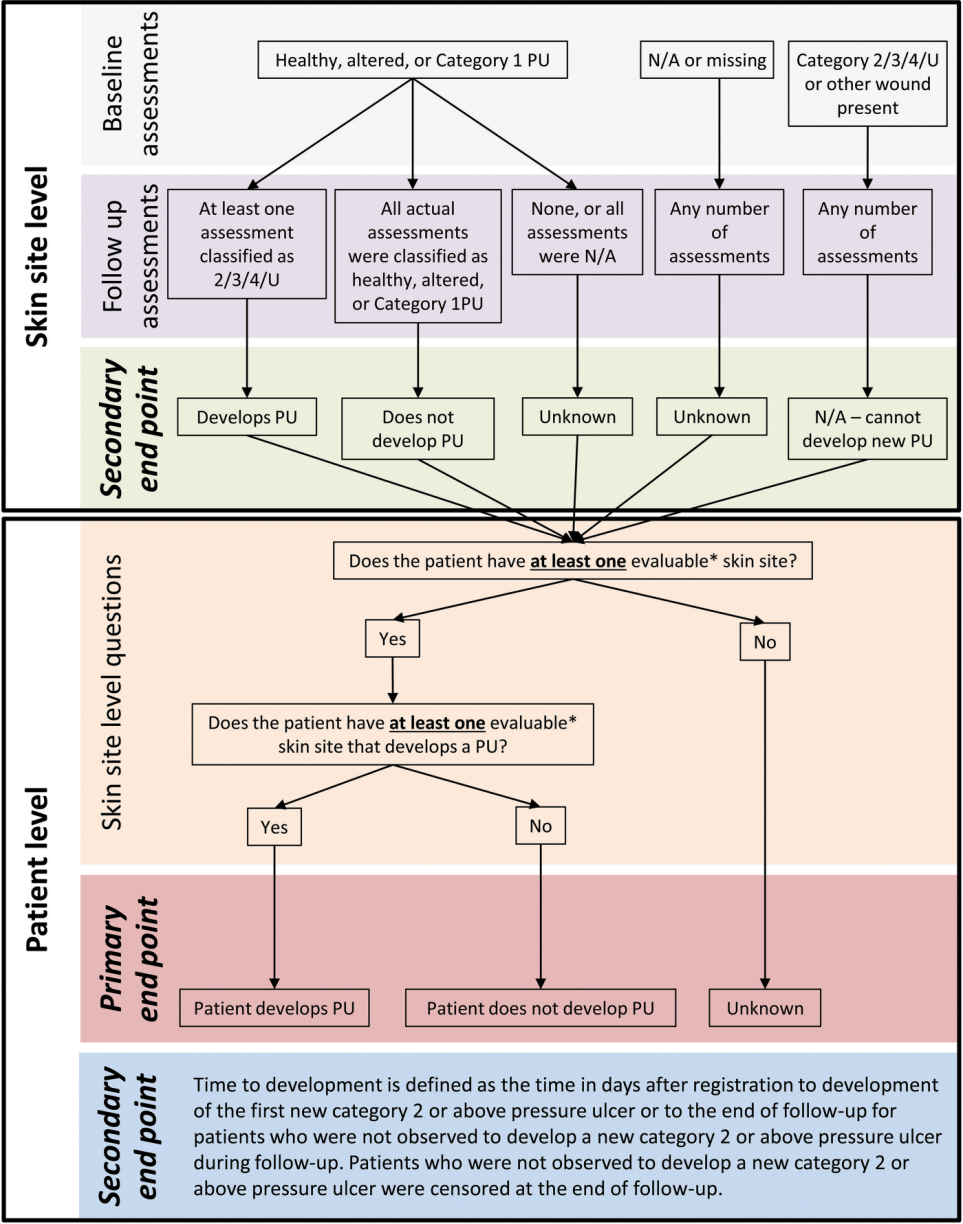
Derivation of skin site level and patient level outcomes. *Evaluable skin sites are such that skin site level outcome can be determined as ‘Develops PU’ or ‘Does not develop PU’. Note that patients had up to nine assessments in total (baseline and twice weekly for up to 30 days). PU, pressure ulcer.

The primary outcome was the development of a new category ≥2 PU. This was defined on a skin site level basis before being combined into a patient level outcome (figure 1). Secondary outcomes were time to development of the first PU and development of a category ≥2 PU at the skin site level.

### Data sources/measurement

Baseline assessment was conducted by trained clinical research nurses and included: demographics (age, gender, setting); a priori risk factors (diabetic status, history of prior weight loss, body mass index (BMI), mattress category, analgesic use, and Braden subdomains)^2^ ^10^ and skin and pain status.

Skin assessment was conducted on 13 prespecified pressure area skin sites (left and right buttocks, heels, elbows, ankles, trochanters, ischial tuberosities and sacrum) and any additional pressure areas identified by the research nurse doing the assessment using the NPUAP/EPUAP PU classification.^9^ PUs were classified as category 1–4 or unstageable. In addition, as general skin condition is predictive of category ≥2 PU development,^2^ ^12^ ^13^ observations of any alteration to intact skin (eg, redness, scar, excoriation, dry, scaly) were recorded and the presence of healthy skin or other wounds was confirmed for each skin site.

Pressure area related pain was defined as pain, soreness or discomfort on any intact pressure area (ie, skin status assessed as normal, altered or category 1).^7^ ^8^ To determine if patients had localised skin pain on any pressure area skin site, they were asked two screening questions with yes or no responses: (1) at any time, do you get pain, soreness or discomfort at a pressure area (prompt: back, bottom, heels, elbows or other as appropriate to the patient)? and (2) do you think this is related to either; your PU OR lying in bed for a long time OR sitting for a long time?^7^ ^8^

Patients were followed up twice weekly for 30 days postregistration or until one of the following criteria applied: (1) no longer at high risk of PU development, (2) transferred to non-participating centre and (3) death.

### Bias

Anonymised screening logs were maintained by centres, and these were monitored to ensure that centres were approaching and recruiting appropriate patients. While blinded end point assessment was not possible, the risk factor and outcome data were recorded by research staff independent of the clinical team.

### Sample size

For risk factor studies using logistic regression, it is recommended that at least 10 patients with the event of interest are required for reliable estimation of effects.^14^ Our primary model planned to include a maximum of 9 factors and therefore required at least 90 patients to develop a new category ≥2 PU. In the absence of prospective data for community-based patient populations,^2^ the sample size estimate was based on previous research in hospital patients,^11^ ^13^ ^15^ suggesting that ∼15% of patients would develop a new category ≥2 PU within 30 days of registration. Therefore, assuming loss to follow-up of 5%, the study required 632 patients.

A further consideration in appraising the sample size estimate was the prevalence of pain at study entry. As no previous work in this field had been undertaken, we considered a range of prevalence rates. We estimated that if we recruited 600 patients (after accounting for 5% loss to follow-up) with 60 (10%) patients having pressure-related pain at baseline, this would allow us to detect a difference of 13.2% between those with and those without pain using a χ^2^ test (80% power, two-sided 5% significance level) assuming 10% of patients without pain and 23.2% of those with pain developed a new PU within 30 days, corresponding to an OR of 2.72 with 95% CI of (1.40 to 5.27). As this was an exploratory study and there was uncertainty around the sample size assumptions, the proportion of patients with pain at baseline and PU incidence was monitored by the statistical team and chief investigator and reported to a subgroup of the Programme Steering Committee.

### Statistical methods

All statistical analyses were carried out in SAS V.9.2 and based on the evaluable patient population. That is, all patients for whom at least one skin site was evaluable (figure 1).

#### Primary analysis: logistic regression model

Univariable logistic regression was conducted to assess a priori variables for inclusion in the multivariable logistic regression model. The a priori variables included the presence of pressure area related pain on a healthy, altered skin or category 1 skin site and other risk factors based on a conceptual framework:^16^ age, diabetes, history of prior weight loss, Braden mobility subscale (completely immobile/very limited mobility vs slightly limited mobility/no limitation with mobility), presence of skin alterations, presence of a category 1 PU and setting (hospital vs community). Candidate variables were considered to be statistically significant and therefore associated with the development of a category ≥2 PU if the p value was <0.1 for the associated likelihood ratio test (LRT). Full stepwise variable selection was conducted to build a multivariable logistic regression model for the odds of developing a category ≥2 PU. Candidate variables were retained in the model if their exclusion led to an increase in deviance with a corresponding p value that was >0.1 for the associated LRT. ORs, corresponding 95% CIs and p values are presented for the univariable and final multivariable models. The level of 0.1 was chosen in line with recommendations for model selection in the analysis of binary data.^17^

Three secondary analyses were conducted as follows.

*Overdispersion model:* Overdispersion model was undertaken to determine if the model could be improved by inclusion of other variables. Further baseline variables (gender, BMI, Braden Scale domains (activity, friction and shear, moisture, nutrition and sensory perception), presence of a category ≥2 PU, presence of chronic wound and type of mattress)^2^ ^16^ were considered for inclusion in the final model from the primary analysis using full stepwise variable selection and the same criteria as for the primary analysis. ORs, corresponding 95% CIs and p values are presented for the additional univariable and final multivariable models.

*Accelerated failure time model*: Full stepwise variable selection was considered for each of the following distributions: Gamma, log-logistic, log-normal and exponential. The model fit of the final models obtained for each distribution were explored using Cox-Snell residual plots. The Gamma distribution was observed to be the most appropriate and so, an accelerated failure time (AFT) model assuming the Gamma distribution was fitted to the data to explore the relationship between the presence of pressure area related pain and time to onset of the first new category ≥2 PU. Univariable analyses were conducted to determine which variables were associated with time to onset of a new category ≥2 PU, and the final AFT model was obtained using full stepwise variable selection with the same criteria as for the primary analysis. Acceleration factors, corresponding 95% CIs and p values are presented for the additional univariable and final multivariable models.

*Skin site level model:* Nesting of skin sites within patients was taken into account using a two-level random-intercept logistic regression model. Univariable analyses and full stepwise variable selection were conducted using the same selection criteria as for the primary analysis to obtain the final multivariable model. For this multilevel model, only evaluable skin sites (figure 1) were analysed, and the variables considered for inclusion in the model were modified to reflect the structure of the data such that age, diabetic status, history of prior weight loss, Braden mobility, setting and analgesic use continued to be patient level risk factors. The presence of pressure area related pain at baseline was assessed for each skin site and was therefore included as a skin site level risk factor, and the presence of skin alterations and the presence of a category 1 PU were combined into a single skin site level risk factor (healthy/skin alterations/category 1 PU). ORs, corresponding 95% CIs and p values are presented for the univariable and final multivariable models.

### Quantitative variables

Age was considered twice for inclusion in the model as a categorical variable (<65 years/65–74 years/75–84 years/≥85 years) and as a continuous variable. The Braden mobility subscale was dichotomised (completely immobile/very limited mobility vs slightly limited mobility/no limitation with mobility), and all remaining variables for inclusion in the primary model were considered to be binary variables. For the secondary analysis model, BMI was considered for inclusion as a continuous variable, the remaining Braden subscales were considered on an ordinal scale, mattress type was categorised as dynamic high-risk pressure-relieving/static high-risk pressure-relieving/non-pressure relieving and the remaining variables for inclusion in the secondary analysis model for the primary outcome were considered to be binary variables.

## Results

### Patients

**Figure 2:**
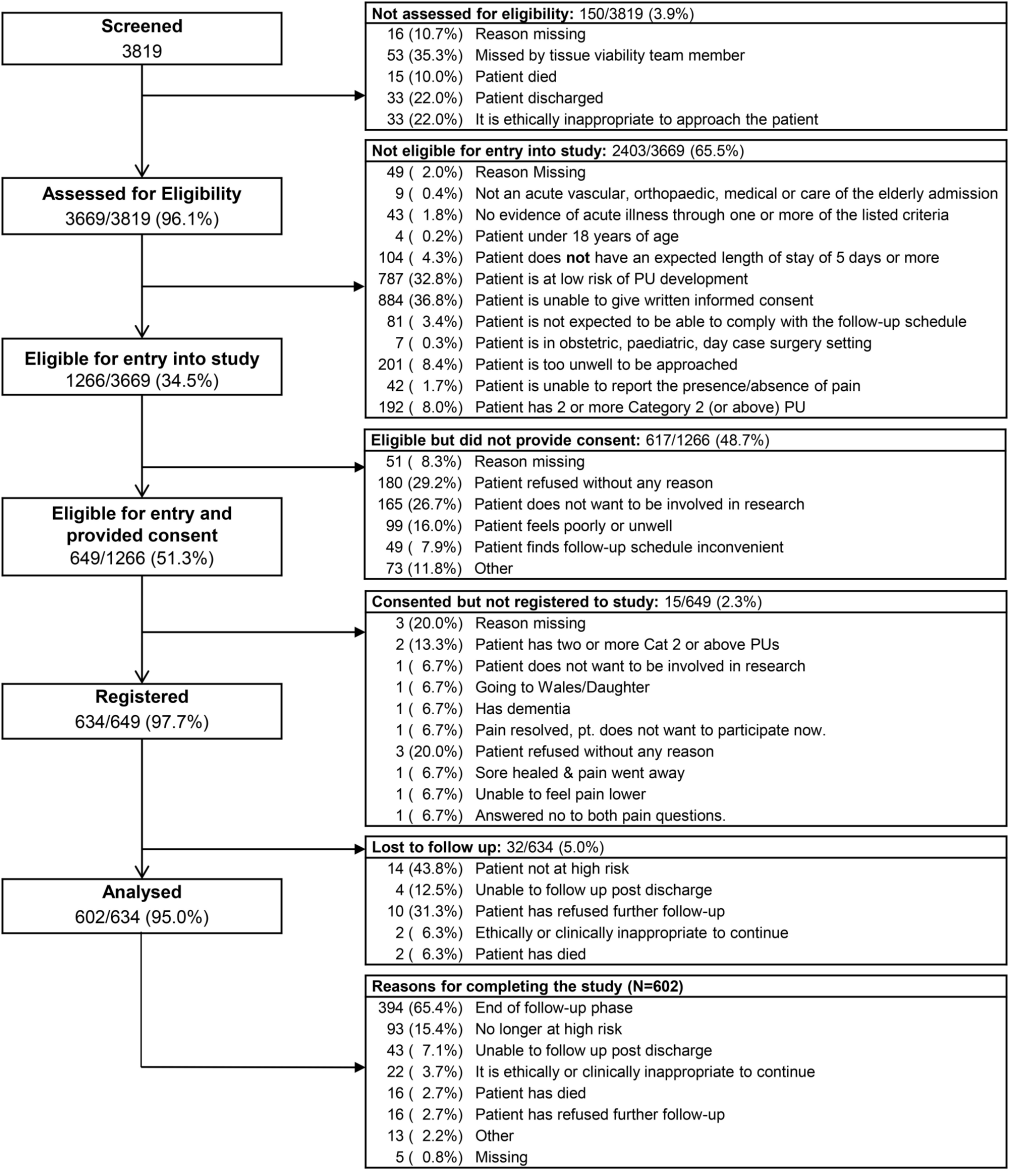
Patient flow.

A total of 3819 patients were screened for participation in the study. A total of 1266 were eligible and of these 634 were registered between 26 October 2009 and 17 November 2011. Reasons for ineligibility and refusals/non-registration are detailed in figure 2. Thirty-two (5.0%) patients did not have any follow-up assessments, and the analysis population therefore comprised 602 evaluable patients (figure 2), including 397 (65.9%) hospital and 205 (34.1%) community evaluable patients. Baseline characteristics are detailed in table 1. The median (range) length of follow-up was 27 (1–34) days.

**Table 1:** Patient level baseline characteristics by setting

Variable	Hospital (N=397)	Community (N=205)	Total (N=602)
Specialty or place assessed			
Vascular	42 (10.6%)	0 (0.0%)	42 (7.0%)
Orthopaedic	155 (39.0%)	0 (0.0%)	155 (25.7%)
Medical	90 (22.7%)	0 (0.0%)	90 (15.0%)
Elderly	32 (8.1%)	0 (0.0%)	32 (5.3%)
Medical/elderly	78 (19.6%)	0 (0.0%)	78 (13.0%)
Patient's own home	0 (0.0%)	49 (23.9%)	49 (8.1%)
Nursing home	0 (0.0%)	27 (13.2%)	27 (4.5%)
Residential home	0 (0.0%)	18 (8.8%)	18 (3.0%)
Rehabilitation unit	0 (0.0%)	104 (50.7%)	104 (17.3%)
Other place assessed	0 (0.0%)	7 (3.4%)	7 (1.2%)
Age			
Mean (SD)	75.6 (12.9)	80.7 (11.7)	77.3 (12.7)
Median (range)	79 (21, 101)	83 (30, 100)	80 (21, 101)
Male	156 (39.3%)	77 (37.6%)	233 (38.7%)
Caucasian	396 (99.7%)	205 (100.0%)	601 (99.8%)
Diabetic	98 (24.7%)	55 (26.8%)	153 (25.4%)
N missing	0 (0.0%)	1 (0.5%)	1 (0.2%)
History of prior weight loss	95 (23.9%)	52 (25.4%)	147 (24.4%)
N missing	0 (0.0%)	1 (0.5%)	1 (0.2%)
BMI			
Mean (SD)	27.1 (9.3)	26.0 (9.9)	26.7 (9.5)
Median (range)	25 (11, 94)	24 (11, 111)	25 (11, 111)
N missing	12 (3.0%)	12 (5.9%)	24 (4.0%)
Chronic wound at baseline	75 (18.9%)	52 (25.4%)	127 (21.1%)
N missing	0 (0.0%)	1 (0.5%)	1 (0.2%)
Worst skin status at baseline			
Healthy	45 (11.3%)	25 (12.2%)	70 (11.6%)
Alterations to intact skin	99 (24.9%)	55 (26.8%)	154 (25.6%)
Category 1	137 (34.5%)	77 (37.6%)	214 (35.5%)
Category 2	98 (24.7%)	42 (20.5%)	140 (23.3%)
Category 3	10 (2.5%)	3 (1.5%)	13 (2.2%)
Category 4	2 (0.5%)	2 (1.0%)	4 (0.7%)
Unstageable	6 (1.5%)	1 (0.5%)	7 (1.2%)
Analgesic in use	366 (92.2%)	182 (88.8%)	548 (91.0%)
N missing	0 (0.0%)	0 (0.0%)	0 (0.0%)
Braden mobility			
Completely immobile	9 (2.3%)	12 (5.9%)	21 (3.5%)
Very limited	204 (51.4%)	58 (58.3%)	262 (43.5%)
Slightly limited	142 (35.8%)	89 (43.4%)	231 (38.4%)
No limitation	42 (10.6%)	46 (22.4%)	88 (14.6%)
Mattress type			
Non-pressure relieving	5 (1.3%)	31 (15.1%)	36 (6.0%)
Static pressure-relieving	168 (42.3%)	105 (51.2%)	273 (45.3%)
Dynamic pressure-relieving	224 (56.4%)	68 (33.2%)	292 (48.5%)
Missing	0 (0.0%)	1 (0.5%)	1 (0.2%)

### Descriptive data

A total of 152 (25.2%) patients developed 223 new category ≥2 PUs during follow-up. There were 464 (77.1%) patients with pressure area related pain on at least one healthy, altered or category 1 skin site at baseline; 28.0% (N=130) of these developed a new category ≥2 PU compared to 15.9% (N=22) of those without pain at baseline (table 2). The summary of worst skin status indicates that a total of 532 (88.4%) patients had some skin damage at baseline.

**Table 2:** Development of PU on a patient level by skin and pain status

Variable	Develops new PU (N=152)	Does not develop new PU (N=450)	Total (N=602)
Presence of skin alterations at baseline			
Yes	109 (29.2%)	264 (70.8%)	373 (62.0%)
No	43 (18.8%)	186 (81.2%)	229 (38.0%)
Presence of category 1 PU at baseline			
Yes	105 (36.2%)	185 (63.8%)	290 (48.2%)
No	47 (15.1%)	265 (84.9%)	312 (51.8%)
Presence of pain at a healthy, altered or category 1 PU skin site			
Yes	130 (28.0%)	334 (72.0%)	464 (77.1%)
No	22 (15.9%)	116 (84.1%)	138 (22.9%)

PU, pressure ulcer.

The analysis population of 602 patients had a combined total of 7863 potential skin sites assessed corresponding to 13 prespecified skin sites for each of the 602 patients and 37 additional ‘other’ skin sites. The majority (77.5%) of skin sites were observed as being healthy skin sites, and pain was reported more frequently with severity of skin status (table 3); for example, 63.1% (351/556) of category 1 skin sites were observed to have pain compared to 40.3% (342/849) of skin sites with alterations and 6.4% (390/6096) of healthy skin sites. After excluding 172 skin sites with an existing category ≥2 PU, 183 skin sites with no baseline assessment (eg, amputation or bandage in situ), 7 assessed as another wound and 18 with no follow-up assessment, 7483 (95.2%) skin sites were evaluable in terms of obtaining a skin site level outcome and were therefore considered for the skin site level analysis.

**Table 3:** Baseline skin and pain assessment on a skin site level basis

Skin classification	Pain yes N (%)	Pain no N (%)	Missing N (%)	Total N (%)
Healthy skin	390 (6.4%)	5700 (93.5%)	6 (0.1%)	6096 (77.5%)
Skin alterations	342 (40.3%)	504 (59.4%)	3 (0.4%)	849 (10.8%)
Category 1	351 (63.1%)	205 (36.9%)	0 (0.0%)	556 (7.1%)
Category 2	116 (78.4%)	32 (21.6%)	0 (0.0%)	148 (1.9%)
Category 3	13 (100%)	0 (0.0%)	0 (0.0%)	13 (0.2%)
Category 4	3 (75.0%)	1 (25.0%)	0 (0.0%)	4 (0.1%)
Unstageable	6 (85.7%)	1 (14.3%)	0 (0.0%)	7 (0.1%)
Unable to assess	6 (7.6%)	52 (65.8%)	21 (26.6%)	79 (1.0%)
Not applicable	2 (2.3%)	26 (29.9%)	59 (67.8%)	87 (1.1%)
Other wound	0 (0.0%)	6 (85.7%)	1(14.3%)	7 (0.1%)
Classification missing	0 (0.0%)	0 (0.0%)	17 (100%)	17 (0.2%)
Total	1229 (15.6%)	6527 (83.0%)	107 (1.4%)	7863 (100.0%)

NB: 602 patients. Each patient had 13 skin assessments and there were 37 ‘other’ sites assessed. The overall total therefore corresponds to (602×13)+37=7863 skin assessments.

Of the evaluable skin sites, a total of 223 (3.0%) developed a new category ≥2 PU. The proportion of skin sites developing a new category ≥2 PU appears to increase with severity of baseline skin status with 1.1% of healthy skin sites, 6.4% of altered skin sites and 18.2% of category 1 PU skin sites observed to develop new category ≥2 PUs (table 4). There were 1077 (14.4%) skin sites with pressure area related pain at baseline and 10.3% of these developed a new category ≥2 PU compared to 1.7% of the skin sites without pressure-related pain (table 4).

**Table 4:** Development of category ≥2 PU by pain and skin assessments on a skin site level basis

Variable	Develops new PU (N=223)	Does not develop new PU (N=7260)	Total (N=7483*)
Pain			
Pain yes	111 (10.3%)	966 (89.7%)	1077 (14.4%)
Pain no	112 (1.7%)	6294 (98.3%)	6406 (85.6%)
Skin status			
Healthy skin	68 (1.1%)	6014 (98.9%)	6082 (81.3%)
Alterations to intact skin	54 (6.4%)	792 (93.6%)	846 (11.3%)
Category 1	101 (18.2%)	454 (81.8%)	555 (7.4%)

*Note that 172 skin sites with an existing Category ≥2 PU, 183 skin sites with no baseline assessment (eg, amputation or bandage in situ) and 25 with no follow-up assessment were excluded leaving a total of 7483 skin sites for analysis.

PU, pressure ulcer.

### Logistic regression model

In the univariable analysis, diabetic status, presence of skin alterations, presence of category 1 and pressure area related pain were statistically significantly associated with the odds of developing a new category ≥2 PU (table 5). Following full stepwise variable selection, the final model (602 patients) indicates that the odds of developing a category ≥2 PU were increased for the following variables: category 1 PU (OR 3.25(95% CI (2.17 to 4.86)), p<0.0001), presence of skin alterations (OR (95% CI)=1.98(1.30 to 3.00), p=0.0014) and presence of pressure area related pain (OR (95% CI)=1.56 (0.93 to 2.63), p=0.0931) (table 5).

**Table 5:** Primary analysis (patient level): logistic regression models

Covariate	OR	95% CI	p Value
*Univariable analyses*			
** **Age (continuous)	1.01	0.99 to 1.022	0.3936
** **Age (categorical) (reference=‘less than 65 years’)			
85 years or older	1.31	0.73 to 2.35	0.4192
65–74 years	1.20	0.63 to 2.29	0.3885
75–84 years	1.17	0.65 to 2.08	0.2996
Diabetic status* (yes vs no)	1.45	0.97 to 2.18	**0.0722**
History of weight loss^†^ (yes vs no)	1.04	0.68 to 1.60	0.8462
Braden mobility (1 or 2 vs 3 or 4)	1.21	0.84 to 1.76	0.3055
Skin alterations (yes vs no)	1.79	1.20 to 2.66	**0.0045**
Category 1 PU (yes vs no)	3.20	2.63 to 4.74	**<0.0001**
Setting (hospital vs community)	0.92	0.62 to 1.35	0.6576
Analgesic use (yes vs no)	0.87	0.46 to 1.62	0.6542
Pressure-related pain on healthy, altered or category 1 skin site (yes vs no)	2.05	1.25 to 3.38	**0.0047**
*Final multivariable model from primary analysis*			
Category 1 PU (yes vs no)	3.25	2.17 to 4.86	**<0.0001**
Skin alterations (yes vs no)	1.98	1.30 to 3.00	**0.0014**
Pressure-related pain on healthy, altered or Category 1 skin site (yes vs no)	1.56	0.93 to 2.63	**0.0931**

Bold values indicate statistical significance. *Missing diabetic status set to ‘no’ for one patient.

†Missing history of prior weight loss status set to ‘no’ for one patient.

PU, pressure ulcer.

### Overdispersion analysis

Univariable analyses of additional variables indicated that gender, Braden moisture, Braden activity, presence of a chronic wound and presence of pre-existing category 2 PU were statistically significantly associated with the odds of developing a new category ≥2 PU (table 6).

**Table 6:** Overdispersion analysis (patient level): logistic regression models

Covariate	OR	95% CI	p Value
*Univariable analyses*			
Gender (female vs male)	0.72	0.49 to 1.04	**0.0780**
BMI (continuous)	1.00	0.99 to 1.02	0.6702
Braden sensory (reference=No impairment)			
Slightly Limited	0.92	0.54 to 1.58	0.6886
Very Limited	0.41	0.05 to 3.40	
Braden Moisture (reference=Rarely moist)			
Occasionally moist	1.83	1.22 to 2.74	**0.0277**
Very moist	1.11	0.51 to 2.44	
Constantly moist	2.39	0.39 to 14.55	
Braden activity (reference=Bedfast)			
Chairfast	1.83	1.05 to 3.19	**0.0775**
Walks occasionally	1.43	0.77 to 2.65	
Walks frequently	0.75	0.23 to 2.40	
Braden nutrition (reference=Excellent)			
Adequate	0.93	0.59 to 1.47	0.6058
Probably inadequate	1.26	0.77 to 2.06	
Very poor	1.10	0.37 to 3.23	
Braden friction and shear (reference=No apparent problem)			
Potential problem	1.06	0.62 to 1.82	0.8036
Problem	1.22	0.64 to 2.36	
Mattress category (reference=dynamic high risk pressure relieving			
Static risk pressure relieving	1.28	0.87 to 1.87	0.2430
Non-pressure relieving	0.95	0.41 to 2.17	
Chronic wound (yes vs no)	1.89	1.24 to 2.88	**0.0032**
Category ≥2 PU (yes vs no)	1.70	1.15 to 2.53	**0.0083**
*Final multivariable model from secondary overdispersion analysis*			
Category 1 PU (yes vs no)	3.20	2.11 to 4.85	**<0.0001**
Skin alterations (yes vs no)	1.90	1.24 to 2.91	**0.0032**
Pressure-related pain on healthy, altered or category 1 skin site (yes vs no)	1.85	1.07 to 3.20	**0.0271**
Category 2 PU (yes vs no)	2.09	1.35 to 3.23	**0.0009**
Braden activity: Chairfast vs Bedfast	1.86	1.03 to 3.36	**0.0476**
Braden activity: Walks occasionally vs Bedfast	1.19	0.62 to 2.29	
Braden Activity: Walks frequently v Bedfast	0.71	0.21 to 2.46	
Chronic wound (yes vs no)	1.66	1.06 to 2.62	**0.0277**

Bold values indicate statistical significance. BMI, body mass index; PU, pressure ulcer.

The final overdispersion model retained the variables (category 1 PU, skin alterations and the presence of pressure-related pain) from the final primary analysis model and the presence of pre-existing category 2 PU (OR (95% CI)=2.09 (1.35 to 3.23), p=0.0009), presence of chronic wound (OR (95% CI)=1.66 (1.06 to 2.62), p=0.0277) and Braden Activity subscale (p value for overall analysis of effects=0.0476). Within this model, the estimate of the odds of category ≥2 PU development in the presence of pressure area related pain increased (OR (95% CI)=1.85 (1.07 to 3.20), p=0.0271) (table 6).

### Accelerated failure time model

The univariable AFT analyses indicated that age, presence of skin alterations, Braden mobility, presence of category 1 PU and presence of pressure area related pain were statistically significantly associated with increased rate of category ≥2 PU development (table 7).

**Table 7:** Analysis of time to PU development (patient level): Accelerated Failure Time models

Covariate	Ratio* of time to developing new Category ≥2 PU	95% CI	p Value
*Univariable analyses*			
Age (continuous)	1.01	1.00 to 1.03	**0.0354**
Skin alterations (yes vs no)	1.40	0.99 to 1.97	**0.0593**
Analgesic use (yes vs no)	1.05	0.61 to 1.83	0.8577
Braden mobility (1 or 2 vs 3 or 4)	1.37	1.00 to 1.87	**0.0498**
Category 1 PU (yes vs no)	2.63	1.93 to 3.58	**<0.0001**
Diabetic (yes vs no)	1.24	0.85 to 1.80	0.2591
History of prior weight loss (yes vs no)	0.90	0.62 to 1.30	0.5715
Pain (yes vs no)	2.68	1.86 to 3.86	**<0.0001**
Setting (Hospital vs community)	1.13	0.81 to 1.58	0.4652
*Final multivariable AFT model*			
Category 1 PU (yes vs no)	2.32	1.73 to 3.12	<0.0001
Pressure-related pain (yes vs no)	2.28	1.59 to 3.27	<0.0001

Bold values indicate statistical significance. Number in final model=602.

*Ratio corresponds to the acceleration factor.

PU, pressure ulcer.

The final AFT model included presence of a category 1 PU (AF (95% CI)=2.32 (1.73 to 3.12), p<0.0001) and presence of pressure area related pain (AF (95% CI)=2.28 (1.59 to 3.27), p<0.0001). The model indicates that patients are likely to develop a category ≥2 PU 2.32 times faster compared to patients who do not have a category 1 PU (AF (95% CI)=2.32 (1.73 to 3.12)). In addition, patients with pressure area related pain are likely to develop a category ≥2 PU 2.28 times faster (95% CI (1.59 to 3.27)) compared to patients who do not have pressure-related pain.

### Skin site level model

The univariable analyses indicated that skin status and the presence of pain were statistically significantly associated with the odds of developing a category ≥2 PU at the same skin site (table 8).

**Table 8:** Skin site level analyses: multilevel logistic regression models

Covariate	OR	95% CI	p Value
*Univariable analyses*			
Age (continuous)	1.01	0.99 to 1.02	0.4808
Diabetic status* (no vs yes)	0.81	0.54 to 1.21	0.3070
History of weight loss† (no vs yes)	1.03	0.68 to 1.57	0.8914
Braden mobility (1 or 2 vs 3 or 4)	1.14	0.80 to 1.64	0.4714
Skin status (reference=healthy skin)			
Skin alterations	6.29	4.21 to 9.40	**<0.0001**
Category 1 PU	27.34	18.5 to 40.4	**<0.0001**
Setting (hospital vs community)	0.91	0.63 to 1.32	0.6148
Analgesic use (no vs yes)	1.33	1.74 to 2.39	0.3350
Pressure-related pain on healthy, altered or Category 1 skin site (yes vs no)	8.68	6.30 to 11.97	**<0.0001**
*Final multivariable model*			
Skin status (reference=healthy skin)			
Skin alterations	4.65	3.01 to 7.18	**<0.0001**
Category 1 PU	17.30	11.09 to 27.00	**<0.0001**
Pressure-related pain on healthy, altered or Category 1 skin site (yes vs no)	2.25	1.53 to 3.29	**<0.0001**

Bold values indicate statistical significance. *Missing diabetic status set to ‘no’ for one missing patient.

†Missing history of weight loss status set to ‘no’ for one missing patient.

PU, pressure ulcer.

The final multivariable multilevel logistic regression model obtained after full stepwise variable selection indicates that skin status and the presence of pressure-related pain are predictive of PU development at the same skin site, after adjusting for patient variation, that is the clustering of skin sites within patients: presence of skin alterations (OR (95% CI)=4.65 ((3.01 to 7.18), p<0.0001, compared to healthy skin), presence of a category 1 PU (OR (95% CI)=17.30 (11.09 to 27.00), p<0.0001, compared to healthy skin) and presence of pressure area related pain (OR (95% CI)=2.25 (1.53 to 3.29), p<0.0001).

## Discussion

### Statement of principal findings

This is the first risk factor study to investigate pain as a risk factor for PU development and found that pain was an independent predictor of category ≥2 PU development in high risk hospital and community patients with acute illness. There was significant evidence that the presence of pressure area related pain is an independent predictor for developing a category ≥2 PU, after adjusting for skin status at baseline, across all four multivariable models. On a patient level, there was marginal evidence that the presence of pain increased the odds for developing a category ≥2 PU by 30 days of follow-up (primary end point), and significant evidence that the presence of pressure area related pain also accelerates the time to developing a category ≥2 PU, after adjusting for other important covariates. On a skin site level, presence of pain is a predictor for developing a category ≥2 PU on the same skin site by 30 days of follow-up, after adjusting for skin status and between patient variation. Other risk factors that emerged include the presence of a category 1 PU and alterations to intact skin at baseline, which is consistent with previous studies which have included category 1 (or equivalent and other skin status variables in multivariable modelling).^2^ ^11^ ^15^ ^18^ ^19^ An unexpected finding was the high proportion of patients (77.1%) who reported pain at baseline; while a number of these patients also had category 1 PUs, the extent of the problem was underestimated in our sample size estimate.

### Strengths and weaknesses of the study

The study was designed to incorporate key quality criteria for the conduct and reporting of risk factor/prognostic factor studies^20–28^ to promote generalisability and minimise bias, including an a priori sample size estimate, risk factors informed by a conceptual framework, risk factor and outcome assessment by a trained clinical research team independent of the clinical team. The majority of patients received the recommended National Institute for Health & Care Excellence (NICE) standard mattress provision of either high specification foam or alternating pressure mattresses^29^ and members of the research team did not alter standard care provision. Limitations of the study included a lack of blinded outcome assessment, which could have been achieved if baseline and follow-up assessments had been undertaken by two different research nurses, but there was not funding or capacity within the clinical research teams for this approach. It is feasible that they could have introduced bias to outcome assessment. The feasibility of using photography for independent blind outcome assessment is currently being determined as part of the HTA PRESSURE2 Trial (http://medhealth.leeds.ac.uk/info/423/skin/1717/pressure_2). It is acknowledged that the patient population is not representative of the general NHS population through exclusion of patients who had cognition problems, were very sick or were terminally ill (figure 2). However, as pain is a symptom of underlying inflammation and/or nerve damage, we consider that the results are generalisable to the wider population. In addition, efforts to assess pain, soreness and discomfort should be made for all patients, including those with cognition problems using pain assessment methods established for this group of patients.^30–32^ It is acknowledged that patients with darkly pigmented skin were under represented in the study.

### Strengths and weaknesses in relation to other studies

Building on our previous research^11^ ^15^ ^33^ ^34^ and in our attempts to maximise the potential event rate and so minimise the required sample size, the PU incidence rate of 25.2% was higher than predicted through inclusion of patients with evidence of acute illness and at ‘high risk’ of PU development. The incidence rate is comparable to other reports of category ≥2 PU incidence. In our systematic review of risk factor studies,^2^ 19 studies reported incidence rates of grade/stage ≥2 ranging from 10.1% to 45.7% in heterogeneous patient populations. Of importance in terms of generalisability was our recruitment of community patients. With the exception of nursing home populations, we found only one risk factor cohort study involving community patients which reported a low incidence rate of 3.2% (55/1567) of stage 2 PUs, but the study did not target patients with evidence of acute illness.^2^ ^35^ The observed incidence rate in the community patient population was 26.3%, although it is noteworthy that the majority of ‘community’ patients were recruited from rehabilitation units, with small numbers recruited in the home. In terms of risk factors identified, results are consistent with findings of the PU risk factor studies which typically identify immobility limitations, skin status and/or perfusion in multivariable analysis.^2^ The patient population is heterogeneous in terms of healthcare setting, but homogenous in terms of the presence of acute illness, with a high proportion of patients with diabetes (25.4%) and an aged population (median (range)=80 (21–101) years) compared to other risk factor studies and indicators of high levels of comorbid disease including chronic wounds (21.1%) and existing category ≥2 PUs (27.2%), maximising the generalisability of the study findings to those patients where primary and secondary prevention interventions require targeting (NICE 2005). It could be argued that the study should have excluded patients with existing category 1 or category ≥2 PUs, but the inclusion criteria reflect the clinical population whereby patients with existing pressure injury are at risk of deterioration or PU development on other skin sites. The study is the first to undertake a skin site level analysis;^2^ this type of analysis allows for assessment of risk factors at patient level, for example diabetes, and at skin site level such as presence of pain at the same skin site. In previous PU research, sample size considerations for multivariable analyses have not been used to inform study design and only 17 studies identified in a systematic review fulfilled the ‘rule of thumb’ sample size estimate of 10 events (or PUs) per variable in the multivariable model.^2^ ^20^ ^23^ ^26^ Of those only seven fulfilled key quality criteria^20–28^ including loss to follow-up of <20%; sufficient presentation of data to assess the adequacy of the method and analysis and a clear strategy for model building (variables included and based on a conceptual framework) and the selected model was adequate for the design.^12^ ^19^ ^34^ ^36–39^

### Meaning of the study: possible explanations and implications for clinicians and policymakers

We have established that the presence of pain increases the risk of category ≥2 PU development and accelerates time to development. This area of practice requires improved assessment, incorporation into risk assessment and treatment strategies to alleviate pain and reduce category ≥2 PU development by pressure relief/reduction. Following these results, the presence of pressure-related pain has been incorporated into the PURPOSE-T a PU risk assessment framework developed as part of the PURPOSE programme (http://medhealth.leeds.ac.uk/info/423/skin/1739/purpose_risk_assessment_framework_raf_work_package).

### Unanswered questions and future research

In our programme of work,^4^ ^6–8^ we have established that pressure area related pain is common in hospital and community patients with intact skin areas and this study which is the first risk factor study to investigate its role suggests that pain is a factor independently predictive of subsequent category ≥2 PU development. This study is the first to undertake a skin site level analysis which allowed skin site-related factors to be taken into account, however further work is required to improve the statistical methodology in this area by using more of the information collected in PU research. Further replication studies are required, and skin site level analyses should be considered in addition to patient level analyses for future PU research.
